# Implementing the care programme for the last days of life in an acute geriatric hospital ward: a phase 2 mixed method study

**DOI:** 10.1186/s12904-016-0102-y

**Published:** 2016-03-05

**Authors:** Rebecca Verhofstede, Tinne Smets, Joachim Cohen, Massimo Costantini, Nele Van Den Noortgate, Luc Deliens

**Affiliations:** End-of-Life Care Research Group, Vrije Universiteit Brussel (VUB) & Ghent University, Brussels, Belgium; Palliative Care Unit, IRCCS Arcispedale S. Maria Nuova, Reggio Emilia, Italy; Department of Geriatrics, Ghent University Hospital, Ghent, Belgium; Department of Medical Oncology, Ghent University Hospital, Ghent, Belgium

**Keywords:** Terminal care, Hospital, Older patients

## Abstract

**Background:**

To improve the quality of end-of-life care in geriatric hospital wards we developed the Care Programme for the Last Days of Life. It consists of 1) the Care Guide for the Last Days of Life, 2) supportive documentation and 3) an implementation guide. The aim of this study is (1) to determine the feasibility of implementing the Care Programme for the Last Days of Life in the acute geriatric hospital setting and (2) to explore the health care professionals’ perceptions of the effects of the Care Programme on end-of-life care.

**Methods:**

A phase 2 mixed methods study according with the MRC framework was performed in the acute geriatric ward of Ghent University Hospital between 1 April and 30 September 2013. During the implementation process a mixed methods approach was used including observation, interviews and the use of a quantitative process evaluation tool. This tool measured the success of implementation using several indicators, such as whether a steering group was formed, whether and how much of the health care staff was informed and trained and how many patients were cared for according to the Care Guide for the Last Days of Life.

**Results:**

The process evaluation tool showed that implementing the Care Programme for the Last Days of Life in the geriatric ward was successful and thus feasible; a steering group was formed consisting of two facilitators, health care staff of the geriatric ward were trained in using the Care Guide for the Last Days of Life which was subsequently introduced onto the ward and approximately 57 % of all dying patients were cared for according to the Care Guide for the Last Days of Life.

With regard to health care professionals’ perceptions, nurses and physicians experienced the Care Guide for the Last Days of Life as improving the overall documentation of care, improving communication among health care staff and between health care staff and patient/family and improving the quality of end-of-life care. Barriers to implementing the Care Programme for the Last Days of Life successfully are, among others, difficulties with the content of the documents used within the Care Programme for the Last Days of Life and the low participation rate of physicians in the training sessions and audits.

**Conclusions:**

Results of this mixed methods study suggest that implementing the Care Programme for the Last Days of Life is feasible and that it has favorable effects on end-of-life care as reported by health care professionals. Based on the identified barriers during the implementation process, we were able to make recommendations for future implementation and further refine the Care Programme for the Last Days of Life before implementing it in a phase 3 cluster randomized controlled trial for the evaluation of its effectiveness.

## Background

Ageing, coupled with a rising prevalence of chronic and degenerative conditions, means that many more older people will need end-of-life care, and this number will continue to increase in future [1]. Although most people wish to die at home [2, 3], a substantial number of older people die within the acute hospital setting, for example in an acute geriatric ward. It is estimated that deaths in institutions such as hospitals are likely to increase in the coming decades [4–6].

Traditionally, high quality care at the end of life has been provided mainly for cancer patients, but optimal end-of-life care should be provided for all patients regardless of diagnosis [1]. Optimal end-of-life care for older hospitalized patients should include good symptom control, respect for patient preferences, appropriate use of diagnostic and therapeutic interventions at the end of life and support for the family [7]. However, many older people dying in hospital experience poor care [8–11]. Research shows that they often receive undesired and burdensome interventions that negatively affect their quality of life [11] and there is also considerable evidence of underassessment and undertreatment of symptoms such as pain [1, 12].

A number of barriers to optimal end-of-life care have been identified including difficulty in recognizing the dying phase, difficulties in withdrawing futile diagnostic procedures and treatments, failure to implement an appropriate end-of-life plan of care, inadequate pain and symptom management and ineffective communication with patients and between patients, relatives and professionals [13–15]. In addition, during medical education, the need for the provision of optimal end-of-life care as part of a physician’s professional duties is insufficiently recognized [14].

**Fig. 1:**
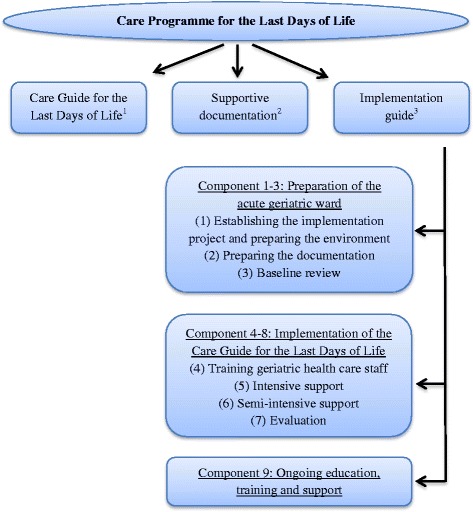
The Care Programme for the Last Days of Life. ^1^ A multi-professional document that provides a template of care for the last days and hours of life with recommendations on different aspects of care and guidance for the psychological and spiritual support of patients and their families. ^2^Supportive documentation consists of a manual for health care staff on how to use the Care Guide for the Last Days of Life, (2) an information leaflet for health care staff about the Care Guide for the Last Days of Life, and (3) three leaflets for family carers about the entering of the dying phase, grief and bereavement and facilities available on the acute geriatric ward. ^3^ This guide assists health care staff in implementing the Care Programme for the Last Days of Life on the geriatric ward during a six-month period and consists of nine components

To improve the quality of end-of-life care for older patients dying in hospital, we developed the Care Programme for the Last Days of Life (hereinafter - Care Programme) for acute geriatric hospital wards [16]. This programme is based on the Liverpool Care Pathway (LCP) programme, taking into account the raised concerns in the UK regarding the LCP. The LCP programme was developed in 1997 in the United Kingdom (UK) and aims to provide a template of multi-professional care for the final days and hours of life and aims to transfer the hospice model of care to mainstream hospital services [17]. The LCP has been widely criticized in the UK since June 2012 for failing to help physicians and nurses provide appropriate care. Raised concerns regarding the LCP arise mainly from inappropriate implementation and use and not the principles of the LCP itself [18]. The Care Programme in Belgium can be considered as being different from the original LCP programme in several ways [16]. It is for instance specifically adapted to the older hospital population and setting. The Care Programme consists of: (1) the Care Guide for the Last Days of Life, (2) supportive documentation and (3) an implementation guide incorporating nine components (Fig. 1; Table 1). The Care Programme aims to introduce and embed the Care Guide for the Last Days of Life (hereinafter - Care Guide), a multi-professional document that provides a template of care for the last days and hours of life in order to ensure that optimal end-of-life care is delivered to every patient dying in an acute geriatric ward.

**Table 1:** Overview of the nine components within the implementation guide

Number	Content
Component 1	Establishing the implementation project and preparing the environment
	▪ Informing the geriatric health care staff about the implementation project and the importance of change in care during the last days of life
	▪ Executive endorsement: acquiring management approval for the trainings and audits ▪ Involvement of specialist palliative care services is recommended: at least one member of the Palliative Support Team of the hospital is member of the steering group
	▪ Facilitators: a nurse and a physician of the geriatric ward
	▪ Formation of steering group: at least four people from the geriatric ward (facilitators included)
	▪ Intensive 2-day training of facilitators
Component 2	Preparing the documentation
	▪ Development of an information leaflet for family carers about the facilities in the geriatric hospital ward
Component 3	Baseline review
	▪ Analyzing end-of-life care data of deceased geriatric hospital patients using the patients’ medical files
Component 4	Training geriatric health care staff
	▪ Feedback of the results to the staff and focusing on improvement within the geriatric ward
	▪ Facilitators and specialist palliative care colleagues train geriatric health care staff with the aid of a training package (i.e. hand-outs with information about the Care Guide for the Last Days of Life, a copy of the Care Guide for the Last Days of Life, a casus to discuss in group etc.)
Component 5	Care Guide use and intensive support
	▪ Care Guide use after sufficient training and education
	▪ Intensive support and supervision by the steering group through repeated coaching, telephone and direct guidance, discussion of clinical cases and clinical audits
Component 6	Semi-intensive support
	▪ Semi-intensive support and supervision by the steering group through repeated coaching, telephone and direct guidance, discussion of clinical cases and clinical audits
Component 7	Evaluation
	▪ To organize a qualitative evaluation of the implementation: evaluating and discussing the performance and progress of each of the previous components
	▪ The qualitative evaluation acknowledges areas where further support, education or training is needed
Component 8	Consolidation
	▪ To adopt a strategy to maintain/improve the implementation and sustainability of the Care Guide
	▪ Support and supervision by the steering group through repeated coaching, telephone and direct guidance, discussion of clinical cases and clinical audits
Component 9	Ongoing education, training and support
	▪ Keeping up to date with developments in end-of-life care and a continuing education and evaluation within the hospital ward

This study aims (1) to assess the feasibility of implementing the Care Programme in the acute geriatric hospital setting and (2) to explore the effects of the Care Programme on end-of-life care according to health care professionals.

## Methods

### Study design

We performed a phase 2 mixed methods study according to the MRC Framework for the development and assessment of complex interventions [19]. The Care Programme was implemented during a six-month period (April-September 2013). In order to assess the feasibility of the implementation process and explore the perceptions of health care professionals of the effects of the Care Programme on end-of-life care, a mixed methods approach was used during the implementation process. This approach included a quantitative process evaluation tool measuring the success of implementation, observation and unstructured interviews to estimate the feasibility of implementation as well as health care professionals’ perceptions of the effects of the Care Programme. The Medical Ethics Commission of the Brussels University Hospital and Ghent University Hospital approved our study proposal which includes taking notes during several meetings, training sessions and personal contacts. Only for the recorded audit we had to gain an additional informed consent of the participants themselves.

### Setting

The Care Programme was implemented in the acute geriatric hospital ward of Ghent University Hospital. At the time of the study, the geriatric ward had 30 beds organized in 17 rooms, run by four geriatricians (including two in training) and 39 nurses and the available support of a hospital-based palliative support team (PST). The PST consists of a palliative care nurse, a social nurse (a combined nurse and social worker), a physician and a clinical psychologist and provides consultation on request and works closely together with existential/spiritual counsellors.

### Data collection

#### Process evaluation tool

We developed a process evaluation tool to measure the success of implementation, i.e. the degree to which each of the nine components of the implementation guide is implemented. In order to know how well each component was implemented, a number of indicators were developed. The indicators, for which an ideal outcome or standard was formulated according to the implementation guide, were measured by the researcher (RV) during the implementation process (Table 2). In this way, the completed process evaluation tool could inform us about the success rate of the implementation process.

**Table 2:** Quantitative and qualitative evaluation of the feasibility of implementing and sustaining the Care Progamme for the Last Days of Life in the geriatric ward of Ghent University Hospital

Component	Quantitative evaluation using the process evaluation tool			Perceived difficulties^a^ among staff in implementing the Care Programme^b^
	Indicator	Standard	Outcome	
1. Establishing the implementation project and preparing the environment	(1) Proportion of health care staff informed about implementation project (%)^c^	100 %	37 % (23/62)	▪ Limited time to establish the implementation project, e.g. composition of steering group with facilitators ▪ Information moment^d^ was organized too early ▪ Information moment did not reach all geriatric health care staff (1) ▪ Content of the 2 day intensive training is not yet fully adjusted to geriatric hospital setting
	(2) Executive endorsement: management approval for organization training and audits	Yes	Yes	
	(3) Composition of steering group	2 nurses 1 physician 1 PST member^e^	2 nurses 1 physician 1 PST member	
	(4) Facilitators: Number Function	≥2 nurse & physician	2 nurse & physician	
	(5) Attendance at the 2 day intensive training by 2 facilitators	Yes	Yes	
2. Preparing the documentation	(6) Development of information leaflet concerning the facilities on the geriatric ward	Yes	Yes	
3. Baseline review^f^	(7) Retrospective evaluation of medical/nursing files of deceased patients	Yes	Yes	▪ Feedback of results to health care staff more feasible if incorporated in training sessions
	(8) Feedback of results to staff	Yes	Yes	
4. Training health care staff on the geriatric ward	(9) Training health care staff			▪ Only feasible if training content is well prepared by steering group ▪ Documents for training health care staff need adaptations (i.e. hand-outs, geriatric casus, manual for using the Care Guide for the Last Days of Life^g^) ▪ Care Guide needs adaptations
	Duration (minutes per edition)	≥90 min	120 min	
	Editions (No.)	≥2 editions	2 editions	
	Nurses involved (%)	100 %	67 % (26/39)	
	Physicians involved (%)	100 %	25 % (1/4)	
5. Use of the Care Guide for the Last Days of Life with intensive support	(10) Introduction of the Care Guide on the ward	Yes	Yes	▪ Training sessions dit not reach enough physicians (9) ▪ No audit was organized (11) ▪ Physicians are hesitant to initiate or use the Care Guide ▪ The term ‘care goal’ lead to misinterpretations and is perceived as being too coercive
	(11) Clinical audit			
	Organized Nurses involved (%) Physicians involved (%)	Yes 100 % 100 %	No No audit organized No audit organized	
6. Use of the Care Guide with semi-intensive support	(12) Clinical audit			▪ Low attendance of health care staff during audit (12) ▪ Diagnosing dying is difficult ▪ Physicians are hesitant to initiate or use the Care Guide ▪ Nurses are too scared of taking responsibility ▪ The term ‘care goal’ is perceived as being too coercive ▪ High workload with double registration
	Organized Nurses involved (%) Physicians involved (%)	Yes 100 % 100 %	Yes 20 % (9/39) 25 % (1/4)	
7. Evaluation^h^	(13) Qualitative evaluation of the implementation	Yes	Yes	
8. Consolidation	(14) Clinical audit			▪ Low attendance of health care staff during second audit (14) ▪ Diagnosing dying is difficult ▪ Physicians are hesitant to initiate or use the Care Guide ▪ Continuing support by all steering group members is important (one nurse of the ward is not sufficient)
	Organized Nurses involved (%) Physicians involved (%)	Yes 100 % 100 %	Yes 26 % (10/39) 0 % (0/4)	
	(15) Proportion of dying patients cared for according to Care Guide during the implementation period (from component 5–8) (%)	≥50 %^i^	57.9 % (11/19)	
9. Use of the Care Guide with ongoing education, training and support	(16) Care Guide still in use on the ward after 1 year	Yes	Yes	The researcher only followed up during the implementation period
	(17) Proportion of dying patients cared for according to Care Guide during the 6 months after completion of implemention period	≥50 %	56.7 % (17/30)	

^a^Perceived difficulties that emerged from the qualitative evaluation

^b^In the further course of this table we used ‘Care Programme’ for the complete term ‘Care Programme for the Last Days of Life’

^c^Health care staff refers to all health carers involved in care on the acute geriatric hospital ward, i.e. nurse, nursing aide, psychologist, physiotherapist, physician, etc

^d^During the information moment, the steering group aims to inform health care staff about the implementation project

^e^One health carer of the Palliative Support Team (PST) should be member of the steering group

^f^To highlight and reinforce the need for change within the ward, the care during the last days of life was retrospectively evaluated by reviewing the medical and nursing files

^g^In the further course of this table we used ‘Care Guide’ for the complete term ‘Care Guide for the Last Days of Life’

^h^The steering group needs to qualitatively evaluate and discuss the performance and progress of each of the previous components in order to identify staff’s training needs and barriers for the use of the Care Guide for the Last Days of Life and provision of optimum end-of-life care

^i^Based on the results of a study performed in the UK and the Netherlands

#### Observation and interviews

A researcher (RV) attended and observed five steering group meetings (meetings of a work group of people coordinating the implementation of the Care Programme), two training sessions and one audit (an evaluation moment led by the steering group where nurses and physicians of the ward reflect upon the use of the Care Guide and specific elements of care delivery; this reflection may help them to identify areas where further education, training or support is needed) related to the implementation and use of the Care Guide. Careful notes were made during each of these meetings and one audit was recorded and transcribed verbatim. A signed informed consent was obtained from each participant attending the audit. The researcher also made notes of face-to-face and telephone contacts with the members of the steering group and of face-to-face contacts with other health care staff of the acute geriatric ward. Finally, the researcher made notes of a meeting of the Advance Care Planning work group, which she attended and during which the Care Programme was discussed. This work group is organized within the Medical Ethics Commission of Ghent University Hospital in order to discuss end-of-life care issues on a regular basis. In total, qualitative data were gathered from twelve nurses, four physicians and two members of the Palliative Support Team i.e. a nurse and a religious counsellor.

### Outcome measures

The first outcome measure, the feasibility of implementing the Care Programme, was assessed using three different methods: the quantitative process evaluation tool, observation and unstructured interviews with health care staff. The quantitative process evaluation tool measured the degree to which the Care Programme was implemented according to the components of the implementation guide, using several indicators. Most important indicators are: the proportion of health care staff informed about the implementation project, the composition of a steering group, number of facilitators, attendance at a two-day intensive training programme by facilitators, retrospective evaluation of end-of-life care and discussion of the results with health care staff, training health care staff in using the Care Guide, introduction of the Care Guide on the geriatric ward, organization of clinical audits and the proportion of patients cared for according to the Care Guide (Table 2).

Observation and unstructured interviews provided additional qualitative data regarding the barriers to the implementation process perceived by the health care staff, which allowed us to gain a deeper understanding of the feasibility of implementing the Care Programme.

The second outcome measure, the perceptions of health care professionals of the effects of the Care Programme on end-of-life care, was explored using observation and unstructured interviews, including notes taken during meetings, verbatim transcription of a clinical audit and notes based on face-to-face and telephone contacts with the members of the steering group and other health care staff.

### Data analysis

#### Quantitative data analysis

The outcomes of the indicators within the process evaluation tool were measured or observed by the researcher (RV). Each outcome was compared with the standard outcome (Table 2).

#### Qualitative data analysis

In order to assess the feasibility of implementing the Care Programme, the textual data, i.e. notes and a transcript, were thematically analyzed. The analysis process consisted of five interconnected stages: (1) involved familiarization, (2) identifying a thematic framework, (3) coding, (4) charting and (5) interpretation [20]. One researcher (RV) performed thematic coding using the nine components within the implementation guide as a framework. A second researcher (TS) checked the coding process and discussed it with RV.

Insights from each set of transcripts served to deepen understanding of the implementation process and to assess the feasibility of the implementation of the Care Programme.

In order to explore the perceptions of health care professionals of the effects of the Care Programme on end-of-life care, thematic analysis was used to capture themes. This analysis was inductive, not restricted by any a priori theoretical framework. After reading the textual data, a preliminary coding framework was developed by one researcher (RV) and discussed with a second researcher (TS). Next, all textual data were read line by line and labels were assigned by one researcher (RV). The coding framework was adjusted where needed, in consensus with a second researcher (TS). The results were discussed within the research team to ensure consistency. A final framework, including results and quotes, was agreed within the research team.

## Results

### Feasibility of implementing the Care Programme

Findings with regard to the feasibility of implementing the Care Programme are presented in Table 2. The results of the process evaluation tool showed that for 15 of the 17 indicators the standard was essentially met; a steering group was formed consisting of two facilitators both of whom attended a two-day intensive training workshop, a leaflet concerning the facilities on the ward was developed, end-of-life care was retrospectively evaluated by reviewing the medical and nursing files and results were subsequently discussed with the staff, the health care staff of the ward were trained in using the Care Guide, two audits were organized and the steering group organized a meeting to evaluate and discuss the performance and progress of the implementation in order to identify training needs and barriers for using the Care Guide and providing optimum end-of-life care. Lastly, more than half of the deceased patients had been cared for according to the Care Guide, i.e. of the 19 people who died on the ward during the implementation period, 11 were cared for according to the Care Guide. Six months after the implementation of the Care Programme, the Care Guide was still in use. In those six months, of the 30 patients died on the ward 17 were cared for according to the Care Guide.

However, despite the fact that two training sessions were organized, only one out of four geriatricians was trained. Furthermore, for two indicators the standard was not met: 37 % instead of 100 % of the health care staff were informed about the implementation project and one out of the three audits that should have been organized was not.

Health care staff identified four types of potential barriers to implementing the Care Programme. Firstly, there were barriers related to practical issues, e.g. many health carers perceived the double registration (i.e. the electronic patient file in combination with the Care Guide in printed version) as a barrier to using the Care Guide. A second type of barrier was related to the content of the documents used within the Care Programme, e.g. some staff had difficulties with the term ‘care goal’ within the Care Guide as it may lead to misinterpretations. According to them the term ‘care goal’ is too coercive; health care staff could perceive the described ‘care goal’ as mandatory for delivering optimal end-of-life care. Hence, it could be that when they would not achieve a ‘care goal’, they would have the feeling that bad end-of-life care was delivered. A third type of barrier was related to the low motivation of some health care staff. Nurses mentioned that low motivation of health care staff resulted in a low participation rate of staff in collective and essential meetings to implement the Care Programme on the geriatric ward. For example, few physicians attended the training sessions and nurses perceived this as an important barrier to introducing and using the Care Guide. A fourth and important barrier was related to difficulties inherent in the organization and provision of care in the last days of life rather than to using the Care Programme. It was for instance found that health care staff had difficulties with recognizing when the dying process had started and, related to this, medical staff often felt resistant to initiating the Care Guide. Furthermore, in relation to the organization of end-of-life care, nurses often felt uneasy about communicating with the physician that a patient had entered the dying phase. They also indicated that they found it difficult to take responsibility for caring for the dying patient according to the Care Guide.

### Health care professionals’ perceptions of the effects of the Care Programme

Four key themes relating to the perceptions of the effects of the Care Programme on end-of-life care emerged from the data analysis. These key themes were: 1) documentation of end-of-life care, 2) content and quality of end-of-life care, 3) communication between health care staff and family carers, and 4) communication among health care staff.

#### Documentation of end-of-life care

Nurses and physicians agreed that there had been an improved documentation of care since the introduction and use of the Care Guide.

*“Previously they said, yes, he is uncomfortable, but, what does that mean? What is uncomfortable? And now you will document it more in detail, it was because of that kind of pain, or it was his breathing, or it was something else”*. [Nurse A]

According to the nurses, the improved documentation of care in the Care Guide also led to a better understanding and delivering of care for the dying patient.

*“For example, if we document the aspiration frequency as four, other caregivers know that we did it four times, I mean that they know, if they are aspirating for the third time, that this is normal”*. [Nurse B]

#### Content and quality of end-of-life care

Since the introduction of the Care Guide nurses felt empowered to approach the patient holistically. Where nurses tended to pay more attention to physical aspects in the care of the dying patient before the introduction of the Care Guide, they claimed they were now more focused on psychosocial and existential/spiritual aspects of care.

*“The Care Guide helps nurses, who were previously focused on the physical aspects, to also think about other care dimensions”.* [Nurse C]

There was a consensus of opinion between the nurses that the Care Guide stimulates them to study each perceived symptom or problem of the dying patient as well as to reflect on an adequate approach to alleviate the symptom.

*“I often hear, people are not comfortable, and then you are wondering what needs to happen, but now the Care Guide has the advantage that this problem is elaborated in depth, that we can find out what the underlying reason is and how we can solve it”.* [Nurse D]

Nevertheless, one nurse was not convinced of the idea that the Care Guide delivers better care for the dying patient. According to her, delivering good symptom control is much more than symptom assessment and reflecting on an ideal approach; a nurse must also be capable of ensuring good symptom control, which is not always the case.

Nurses and physicians also agreed that using the Care Guide ensures better continuity of care at the end of life. Because of the four-hour registration of symptoms (symptoms need to registered every four hours in order to ascertain an optimal symptom management) and reported interventions or actions in the Care Guide, each staff member involved in the care of the dying patient has knowledge of the clinical status of the patient and of the medical and nursing interventions previously taken, which allows them to ensure continuity of care.

*“I think it is very interesting as a communication tool, in the continuity of care, if one nurse takes it over from another nurse, that she clearly sees what already happened and how far we are in the provision of end-of-life care, and what she can further improve during her late shift”*. [Nurse E]

According to some nurses the Care Guide provides a structure in delivering optimal end-of-life care to patients and family carers. One nurse stated:

*“When I start to support a dying patient with the Care Guide, I will do it in a structured way, I will tell him, it is not going well and you will die, and I will consider his spiritual/existential needs and wishes, then I will think about all these issues”*. [Nurse A]

Nurses and physicians agreed that the Care Guide serves as a memory aid as it reminds them to consider all relevant aspects of end-of-life care. Two physicians perceived this as one of the most important advantages of the Care Guide. However, these physicians felt that for them the Care Guide was only an aide-memoire as they believed end-of-life care was already optimal in their ward though in hospitals struggling to deliver good end-of-life care it could be of more benefit.

Reference was also made by several nurses and one physician to medication policy, i.e. anticipatory prescribing and effective medication use. Nurses confirmed that the Care Guide implied a clearer policy regarding anticipatory prescribing of medicines to ensure that there is no delay in responding to symptom if they occur.

*“Previously, before the introduction of the Care Guide, it happened that I had to call the on-call physician during a weekend and ask him to prescribe Morphine, which often resulted in a delayed response to pain”.* [Nurse A]

One respondent mentioned that, since the Care Guide had been introduced, she gives medicines for symptom control only when needed, at the right time and just enough and no more than what is needed to relieve the symptom. However, another respondent expressed concerns about the management of pain medication.

*“Once a patient is supported by the Care Guide, some nurses think they must give and increase all the medication, whereas this is not necessary for every patient, and it must be more tailored to the individual needs and wishes of the patient”*. [Nurse C]

#### Communication between health care staff and family carers

Some nurses agreed that the Care Guide helped them to reflect on practical issues that are important to relatives, for example:

*“It is a control check for us, do I have a phone number of the patient’s family carer, or do I know when I can call this person, or that you are at least reflecting on it”.* [Nurse F]

Nurses agreed that since the introduction of the Care Guide communicating with the relatives had been given a higher priority. According to them the impending death and care of the dying person could be discussed in a more open way. However, the Care Guide did not change the extent of involvement of the patient’s family carer in discussions regarding the plan of care. Some nurses believed that decisions and reasons for them were already communicated and explained well enough to family carers before the introduction of the Care Guide.

*“Actually, what we are doing, we say, yes, it’s a bit…”* [Nurse A] (nurse is hesitant to express her opinion about the use of the Care Guide)

*“I don’t think it’s different than before the introduction of the Care Guide”* [Nurse E]

*“No no”* [Nurse C]

*“No, I think it’s indeed a bit the same as we did before”* [Nurse A]

*“Actually it doesn’t change; from the moment you see that someone’s condition is worse, you communicate that and you say, look, we stop antibiotics”*. [Nurse C]

#### Communication among health care staff

There was an overall agreement among nurses and physicians that communication between health care staff was improved after implementation of the Care Guide. They were convinced that introduction of the Care Guide 1) facilitates discussion between medical and nursing staff about recognizing the dying phase in patients, 2) stimulates hospital staff to inform the patient’s general practitioner about their impending death and 3) creates the opportunity to evaluate and discuss the delivered care between each other.

*“You have to report a lot…”* [Nurse B]

*“Yes, to describe details, so that you can check with your colleague…”* [Nurse G]

*“It will be a little more objective”* [Physician]

*“…why he doesn’t think that…or that you think someone should have more comfort. You’ve got a medium now, in order to evaluate the care commonly”* [Nurse G]

*“Your arguments are more clear for each other”* [Nurse E]

*“So the communication between us improves”* [Nurse G]

## Discussion

The results of this mixed methods study suggest that implementing the Care Programme in the acute geriatric hospital setting is feasible and also has valuable effects on end-of-life care as perceived by health care professionals: nurses and physicians experienced it as improving the overall documentation of care, improving communication among health care staff and between health care staff and patient/family and improving the quality of end-of-life care. However, the indicators informing health care staff about the implementation project, training nurses and physicians and organizing audit(s) were not fully met. Difficulties with the content of the documents used within the Care Programme and the low participation rate of physicians in the training sessions and audits were perceived as important barriers to successful implemention of the Care Programme in the geriatric ward.

The proportion of dying patients cared for according to the Care Guide is an important indicator of the success of implementation in terms of consolidation and ongoing use of the Care Guide. During the implementation period and six months after, approximately 60 % of all patients who died in the geriatric ward were cared for according to the Care Guide. Other studies performed in the UK and the Netherlands found that the LCP, an end-of-life care pathway for the last days of life similar to our Care Guide, had been used for around 85 % of all cancer patients who died during the research period in a hospice, and for 50 % of all cancer patients who died in a Palliative Care Unit [21]. Nevertheless, we deem 60 % as a sufficient result for our study [22, 23], recognizing that the dying phase is considered to be more difficult in older patients who often suffer from multiple chronic conditions than in those dying from cancer as the actual death is often more unexpected [22]. In addition, our mixed methods study was conducted in a hospital setting, where the focus may be more on cure or life-prolonging than in hospices or palliative care units [23]. Furthermore, results from an Italian cluster randomized controlled trial performed in hospitalized cancer patients showed that during LCP implementation only 34 % of dying patients were cared for in accordance with the programme and that this percentage decreased during the six months after implemention [24].

Other factors that indicate that implementing the Care Programme is feasible are that the acute geriatric ward was able to create a steering group, involve palliative care services (e.g. members of a Palliative Support Team), appoint two facilitators responsible for the

coordination of the implementation process, organize training sessions on why and how to use the Care Guide and organize audits.

The health care staff of the geriatric ward perceived the Care Programme as favorable in terms of its positive effects on end-of-life care, which confirms the findings of earlier qualitative studies [25–27]. More specifically, according to nurses and physicians, use of the Care Programme improves the overall documentation of care and positively influences communication among health care staff and between health care staff and patients/families. They also experienced a positive effect of the Care Programme on the quality and content of end-of-life care. For instance, according to nurses and physicians, using the Care Guide stimulates a multidisciplinary approach in care at the end of life. It also stimulates greater reflection among health care staff on end-of-life care, stimulates continuity of care, helps structure care delivery and promotes a clearer policy regarding anticipatory prescribing of medicines. Moreover, as no additional resources or persons were needed to implement the Care Programme, positive effects could be achieved without any additional cost.

Since one nurse remarked that good end-of-life care requires more than just the use of the Care Guide, training of health care staff in symptom management and in delivering optimal end-of-life care is required if we want the Care Guide to add value to end-of-life care. This confirms what was recently recommended by a review performed in the UK in response to the concerns about the LCP [18]. According to that review, the importance of a well thought-out implementation strategy, underpinned by training and education of all staff involved, cannot be overestimated, and should therefore be considered as a priority when implementing the pathway [18].

Barriers to implementing and using the Care Programme in the acute geriatric hospital ward identified in our study include practical issues such as insufficient time, the administrative burden of using the Care Guide and the lack of integration with electronic patient files. The same barriers were identified in a recently published qualitative study about barriers and facilitators to implementation of the LCP in a hospital, a hospice, a home care setting and a nursing home in the Netherlands [28].

Lack of motivation of some health care staff, for instance reflected in the low involvement of physicians during the training sessions, was another important barrier to successful implemention. This lack of motivation may have been related to the insufficient authority and influence of the steering group, which was also identified as an important barrier in the Dutch qualitative study [28].

Finally, the difficulties with recognizing the dying phase and the resistance of health care staff to initiating the Care Guide also seem to be important barriers to adequately use of the Care Guide. This barrier was also identified in a study investigating barriers to implementation of an integrated care pathway for the last days of life in nursing homes [29]. Diagnosing when a patient is dying, understanding the dying process and communicating about dying are indeed very difficult issues in practice, but they are a prerequisite for delivering good end-of-life care.

The barriers identified enabled us to further refine the Care Programme. For instance, the Care Guide and other supportive documents were adapted to overcome the barriers related to the content of the documents. For example, the term ‘care goal’ which was used in the Care Guide was changed into ‘point of attention’; another example is that practical barriers such as a lack of time can be overcome by recommending and predicting more time for preparation within the implementation guide. Furthermore, based on the barriers identified, we were also able to make recommendations for future implementation. Firstly, wards that are willing to implement the Care Programme are encouraged to seek management approval to create more time to compose a steering group and inform all involved health care staff. Secondly, in order to overcome the low motivation of some health care staff and thus the low participation in training and audits, the importance of a very motivated steering group and good facilitators who can enthuse other staff cannot be overestimated as they are key to successful implementation [28]. Thirdly, difficulties inherent to the organization and provision of end-of-life care need to be incorporated into and discussed during the training sessions and audits. It is therefore essential that all health care staff who will use the Care Guide attend these meetings.

Further research in order to gain a better understanding of these barriers and how they could best be approached or addressed would also be very helpful. Allocating specific tasks and time to the facilitators of the geriatric ward as well as introducing an e-health module regarding education and training for nurses and physicians may for example be considered as important for further implementation of the Care Programme.

Over many years various pathways have been developed and implemented in order to improve end-of-life care [30–32]. Since 2012 the LCP has been widely criticized for failing to provide appropriate care and an independent review has recommended that it should be phased out in the UK [18]. However, this should not be a reason to abandon any efforts to structure and further improve end-of-life care in health care settings. Rather, it pinpoints the need to properly develop, evaluate and implement ameliorated end-of-life care improvement programmes taking into account the context in which it will be implemented as well as the concerns raised in the UK, such as improper implementation leading to cases of inadequate end-of-life care [16]. With respect to the raised concerns in the UK the terminology was changed from ‘Pathway’ to ‘Care Guide’ and an implemention guide was developed. This implementation guide incorporates nine components to be performed and includes a detailed and elaborated training package to help health care staff in educating and supporting their colleagues in using the Care Guide in a correct and compassionate way [16]. Additionally, a quantitative process evaluation tool was developed in order to assess and monitor the quality of implementation. Furthermore, a phase 2 study is necessary to identify which factors can result in better or worse implementation in the geriatric hospital setting. It allows us to better understand and monitor the process of implementation and further improve the Care Programme before evaluating its beneficial effects and potential harms in a larger cluster randomized controlled trial [18].

An important strength of our study is that it uses a phase 2 approach according to the MRC Framework for the development of a complex intervention [19]. This framework, following a five phase iterative approach from pre-clinical phase to large-scale implementation, provides a valuable structure to guide the development and modelling of a complex intervention to improve end-of-life care in acute geriatric hospital wards [19]. Secondly, our study uses methodological triangulation [33]; multiple qualitative methods (i.e. notes and a transcripts) and a quantitative method to evaluate the feasibility and effects of the Care Programme.

There are also limitations in the study that need to be considered. Firstly, because it took place only in one geriatric hospital ward, located in a university hospital, our results cannot be generalized to other wards in other hospitals. Secondly, we only explored the perceptions of health care staff whereas family carers and patients could have provided additional information on the effects of the Care Programme.

## Conclusions

Results of this mixed methods study suggest that implementing the Care Programme in an acute geriatric hospital setting is feasible as most of our indicators for a successful implementation were met. Nurses and physicians also perceived the Care Programme as favorable in terms of its positive effects on the documentation of care and the content and quality of end-of-life care and communication among health care staff and between health care staff and patient/family. However, several barriers to the implementation process were perceived relating to practical issues, the content of the supportive documents within the Care Programme, the low involvement of health care staff during meetings and training and difficulties inherent to the organization and provision of end-of-life care in the last days of life. To resolve most of these barriers, adaptations were made to the Care Guide and implementation guide that have resulted in a refined Care Programme. However, barriers related to the low motivation of staff and the organization and provision of end-of-life care are more challenging to resolve. Health care staff desiring to implement and use the Care Programme on their ward should take these challenges into consideration. Further research should focus on gaining a better understanding of the barriers and of how they could best be addressed.

## Abbreviations

LCP: Liverpool Care Pathway
MRC: Medical Research Council
PST: palliative support team
